# 'Fat mass and obesity associated' gene (*FTO*): No significant association of variant rs9939609 with weight loss in a lifestyle intervention and lipid metabolism markers in German obese children and adolescents

**DOI:** 10.1186/1471-2350-9-85

**Published:** 2008-09-17

**Authors:** Timo D Müller, Anke Hinney, André Scherag, Thuy T Nguyen, Felix Schreiner, Helmut Schäfer, Johannes Hebebrand, Christian L Roth, Thomas Reinehr

**Affiliations:** 1 Department of Child and Adolescent Psychiatry and Psychotherapy, University of Duisburg-Essen, Essen, Germany; 2 Institute of Medical Informatics, Biometry and Epidemiology, University of Duisburg-Essen, Essen, Germany; 3 Institute of Medical Biometry and Epidemiology, Philipps-University, Marburg, Germany; 4 Children's Hospital Medical Center, University of Bonn, Germany; 5 Children's Hospital & Regional Medical Center, University of Washington, Seattle, USA; 6 Vestische Kinder- und Jugendklinik, University of Witten/Herdecke, Datteln, Germany

## Abstract

**Background:**

We have previously identified strong association of six single nucleotide polymorphisms (SNPs) in *FTO *(fat mass and obesity associated gene) to early onset extreme obesity within the first genome wide association study (GWA) for this phenotype. The aim of this study was to investigate whether the obesity risk allele of one of these SNPs (rs9939609) is associated with weight loss in a lifestyle intervention program. Additionally, we tested for association of rs9939609 alleles with fasting blood parameters indicative of glucose and lipid metabolism.

**Methods:**

We initially analysed rs9939609 in a case-control study comprising 519 German overweight and obese children and adolescents and 178 normal weight adults. In 207 of the obese individuals who took part in the outpatient obesity intervention program 'Obeldicks' we further analysed whether carrier status of the obesity risk A-allele of rs9939609 has a differential influence on weight loss after the intervention program. Additionally, we investigated in 480 of the overweight and obese patients whether rs9939609 is associated with fasting blood levels of glucose, triglycerides and HDL and LDL-cholesterol. Genotyping was performed using allele specific polymerase chain reaction (ARMS-PCR). For the association study (case-control approach), the Cochran-Armitage trend test was applied. Blood parameters were analysed using commercially available test kits and the log10-transformed blood parameters and changes in BMI-standard deviation scores (BMI-SDS) were analysed by linear regression with sex and age as covariates under an additive mode of inheritance with the rs9939609 A-allele as risk allele.

**Results:**

We confirmed the association of the risk A-allele of rs9939609 with overweight and early onset obesity (one sided p = 0.036). However, we observed no association of rs9939609 alleles with weight loss or fasting levels of blood glucose, triglycerides and cholesterol.

**Conclusion:**

We confirmed the rs9939609 A-allele as a risk factor for early onset obesity whereas its impact on weight loss or on serum levels of glucose, triglycerides and cholesterol could not be detected in our samples.

**Trial Registration:**

**This study is registered **at clinicaltrials.gov (NCT00435734).

## Background

The first genome wide association study (GWA) for early onset extreme obesity recently revealed highly significant association of six strongly linked SNPs in intron one of *FTO *with obesity [1]. One of these genetic variants (rs9939609) had previously been identified by a GWA for type 2 diabetes mellitus (T2DM), revealing that rs9939609 is associated with an increased risk for both T2DM and obesity [2]. However, after adjustment for BMI the association of the at-risk A-allele with T2DM vanished, indicating that the impact of *FTO *on T2DM is mainly due to the association of *FTO *with BMI [2]. The latter result had independently been confirmed in 13 samples comprising 38,759 individuals for which a meta-analysis showed that the A-allele is associated with a 31% increased obesity risk [2]. An association of the rs9939609 A-allele with obesity was also recently found in a GWA based on an adult population-based study group from Sardinia [3]. Additionally, several other SNPs in the intronic region of *FTO*, that are in strong linkage disequilibrium (LD) with rs9939609, showed similar effects on both childhood [1,4] and adult obesity [2-4].

To corroborate the association of this variant with early onset extreme obesity, we initially performed a case control study comprising 519 German overweight and obese children and adolescents and 178 normal weight adults. In a follow-up analysis we then assessed the association of rs9939609 with weight loss in a subset of 207 German overweight and obese patients who participated in an outpatient obesity intervention program. Very recently, another follow-up study analysing another *FTO *SNP (rs8050136) in 204 obese subjects revealed no impact of this variant on weight loss after an intervention program [5]. As increased metabolic and cardiovascular risk factors (e.g. increased blood levels of glucose, triglycerides and low density lipoprotein (LDL)-cholesterol as well as decreased levels of high density lipoprotein (HDL)-cholesterol) are observable in many but not all obese children and adolescents, we additionally analysed in 480 German obese children and adolescents whether fasting levels of blood glucose, triglycerides and cholesterol (LDL, HDL) are associated with the rs9939609 genotype status at this developmental stage.

## Methods

### Subjects

The ascertainment strategy was previously described in detail [6]. Briefly, the investigated overweight and obese children had no endocrine or syndromal disorders and came to our outpatient centre specialized in paediatric obesity and endocrinology from 1999 to 2005. All individuals were of European descent. The patients were examined with the standardized diagnostic procedure on the basis of the guidelines of the American Academy of Paediatrics. Height was measured to the nearest centimeter with a rigid stadiometer. Weight was measured unclothed to the nearest 0.1 kg with a calibrated balance scale. The degree of overweight was quantified with Cole's least mean square method [7], which normalized the BMI skewed distribution in childhood and expressed BMI as a standard deviation score (BMI-SDS) which is comparable to the BMI z-score as e.g. used by the Center for Disease Control (CDC). Written informed consent was given by all participants and in the case of minors, by their parents. The study was approved by the ethics committees of the Universities of Witten/Herdecke, Bonn and Essen and carried out according to the Declaration of Helsinki.

#### Case-control study

The study group (cases) comprised 519 (249 male) German overweight and obese children and adolescents (mean BMI 28.88 ± 5.23 kg/m^2^, mean age 10.71 ± 3.10 years, median age and gender specific BMI percentile 99.5; 485 patients (93.4%) had a BMI above the 97^th ^percentile). The overweight and obese individuals were either recruited at the Department of Pediatrics, University of Bonn, Germany (n = 186) or at the 'Vestische Kinderklinik Datteln', University of Witten/Herdecke, Germany (n = 333). The control group included 178 (70 male) normal weight adults (mean BMI 21.76 ± 1.08 kg/m^2^, mean age 24.58 ± 2.56 years, median age and gender specific BMI percentile 51). The normal weight individuals were healthy German students who were recruited by the Department of Child and Adolescent Psychiatry, University of Marburg, Germany. None of the individuals used in this study has been used in our previously published GWA for early onset extreme obesity [1].

#### Follow-up study

A subset of 207 overweight and obese individuals (94 males, mean age 10.79 ± 2.52 years, mean BMI 27.89 ± 4.04 kg/m^2^, median age and gender specific BMI percentile 99.2) took part in the outpatient obesity intervention program 'Obeldicks' at the 'Vestische Kinderklinik Datteln', University of Witten/Herdecke, Germany. To participate in the intervention program, the children had to prove their motivation by filling out a questionnaire concerning their eating and exercise habits and by attending exercise groups for overweight children regularly for at least 8 weeks. Only children who had filled out the questionnaires and who had participated in the exercise groups were included in the "Obeldicks" lifestyle intervention program independently of what they have stated in the questionnaires [8].

To analyse whether the rs9939609 AA or AT genotype is associated with decreased weight loss during the intervention program, BMI of these patients was measured before and about 12 months (minimum 10 months) after the beginning of the weight loss therapy. The outpatient intervention program was based on physical exercise, nutritional education, and behaviour therapy including individual psychological care of the child and his or her family. The recommended diet was fat and sugar reduced as compared to the every-day nutrition of German children [9].

### Molecular and genetic methods

#### Genotyping

The *FTO *SNP rs9939609 was genotyped using ARMS-PCR [10]. Primers were derived from genomic entry AC007909.8; rs9939609 F_out_: 5'-TGG CTC TTG AAT GAA ATA GGA TTC AGA A-3'; R_out_: 5'-AGC CTC TCT ACC ATC TTA TGT CCA AAC A-3', F_in_: 5'-TAG GTT CCT TGC GAC TGC TGT GAA TAT A-3', R_in_: 5'-GAG TAA CAG AGA CTA TCC AAG TGC ATC TCA-3' (product size outer primers: 321 bp, T-allele: 178 bp, A-allele: 201 bp). For validity of genotypes, alleles were rated independently by at least two experienced individuals. Discrepancies were resolved unambiguously either by reaching consensus or by retyping. To ensure validity of genotyping, we double-genotyped rs9939609 in 85 individuals of the GWA sample published in [1]; the genotypes were in 100% accordance. These 85 individuals where, however, not incorporated in the analyses.

#### Blood parameters

Fasting blood parameters for glucose and lipid metabolism (blood glucose, triglycerides, LDL and HDL cholesterol) were obtained in up to 480 overweight and obese individuals. Blood samples were taken at baseline in the morning after an overnight fast. Plasma levels of glucose, triglycerides, as well as LDL and HDL cholesterol were measured using commercially available test kits (Roche Diagnostics, Mannheim, Germany; Boehringer, Mannheim, Germany; Ortho Clinical Diagnostics, Neckargemuend, Germany; Abbott, Wiesbaden, Germany). Intra- and inter-assay variations of these variables were less than 5%.

### Statistics

Hardy-Weinberg equilibrium was tested in all independent study groups (exact test). All data were analysed using a (log-) additive genetic model as previously proposed by Frayling et al. [2]. Cochran-Armitage trend test was used for the case-control sample and a one-sided p-value for the previously reported effect was derived [2]. The log10-transformed blood parameters and changes in BMI-standard deviation scores (BMI-SDS and Δ BMI-SDS for changes) were analysed by linear regression with sex and age as covariates under an additive mode of inheritance with the A-allele as risk allele. For analyses of blood parameters and BMI-SDS change, nominal two-sided p-values are presented. Furthermore, 95% confidence intervals for the estimates are provided. A significance level of 5% was applied. Power calculations were done using QUANTO Version 1.2.3 . 519 cases and 178 control pairs were estimated to yield a power of ≥0.95 to detect a multiplicative genotype relative risk of 1.5 (α = 0.05; one-sided for confirmation of the directional hypothesis) assuming a minor allele frequency (MAF) of 0.4. For regression based analyses (207 to 519 individuals), the power estimate ranged between 0.71 and 0.97 to detect a standardized additive effect of 0.25 (α = 0.05; two-sided) again assuming a MAF of 0.4. Thus, both study parts were at least well powered to detect moderate to strong effect sizes. All statistical analyses were performed using either R 2.7.0 or StatXat 5.0.

## Results

The initial case-control study comprising 519 German overweight and obese children and adolescents and 178 normal weight adults confirmed the association of the rs9939609 A-allele with overweight and early onset obesity (one-sided p = 0.036; OR_AT _1.24, 95% CI 0.98–1.57; OR_AA _1.54, 95% CI 0.96–2.46; Table 1). Thus, we independently confirmed the previously described association of this variant with early onset extreme obesity. As a sensitivity analysis we combined the data of our 178 normal weight adults with those of 442 underweight controls who have previously been analysed and reported in our GWA for early onset extreme obesity [1]. Again, the rs9939609 A-allele was strongly associated with early onset obesity (one-sided p = 5.70 × 10^-6^; OR_AT _1.47, 95% CI 1.24–1.73; OR_AA _2.15, 95% CI 1.54–2.98). Note that the latter case-control comparison was only performed to demonstrate the robustness of the initially observed (independent) association. In the follow-up study comprising 207 obese individuals who participated in the obesity intervention program 'Obeldicks', we found no association of the rs9939609 genotype status on body weight loss due to the intervention program (table 2). However, we observed a small but not significant trend for the A-allele carriers to loose slightly less weight than the homozygous T-allele carriers (Δ BMI-SDS AA: 0.27 ± 0.27, AT: 0.25 ± 0.27, TT: 0.33 ± 0.40; 95% CI: 0.091 – 0.0027, p = 0.287, table 2). It is noteworthy that the initially observed association of this variant with obesity was, however, not found in the degree of obesity within the group of these obese individuals. Accordingly, there were no differences in the BMI-SDS within the whole group of obese individuals at baseline (table 3). Also analyses of several fasting blood parameters in up to 480 of the overweight and obese patients revealed no meaningful association of rs9939609 genotypes with serum levels of glucose, triglycerides and LDL and HDL cholesterol (all p > 0.05; Table 3).

**Table 1:** Genotype and allele frequencies of the intronic *FTO *SNP rs9939609

		Genotypes N (%)			Alleles^1^ (%)		Odds ratio^2^ [95% CI]		
				
	N	TT	AT	AA	T	A	TT	AT	AA
Cases^3^	519	140 (27)	238 (46)	141 (27)	0.50	0.50	1.00	1.24	1.54
Controls^4^	178	56 (31)	86 (48)	36 (20)	0.56	0.44		[0.98...1.57]	[0.96...2.46]

^1 ^genotype and allele frequencies of control samples (normal weight adults) are similar to the allele frequencies reported for the European population in the dbSNP database , ^2 ^asymptotic one-sided p-value for association of the A-allele with obesity is p = 0.036, ^3 ^cases comprise 519 German overweight and obese children and adolescents, ^4 ^controls comprise 178 normal weight healthy adults

**Table 2:** Analyses of BMI-SDS changes in German obese children and adolescents who participated in the obesity intervention program 'Obeldicks'

Parameter	N^1^	Genotype	N (%)	Mean ± SD	Additive genetic model^2^		
					
					Estimate	95% CI	p-value^3^
Δ BMI-SDS^4,5^	207	TT	53 (26)	0.33 ± 0.40	-0.032	-0.091...0.027	0.287
		AT	86 (41)	0.25 ± 0.27			
		AA	68 (33)	0.27 ± 0.27			

^1 ^total number of individuals who participated in the obesity intervention program; ^2 ^linear regression analyses for BMI-SDS  including covariates age and sex- including baseline BMI-SDS did not substantially alter the result; the p-value for the sex main effect was 0.740; ^3 ^two-sided p-value; ^4 ^median time interval between repeated measurements 12 months; positive values for descriptive statistics indicate weight reduction in units of BMI-SDS; asymptotic two-sided p-value for Kruskal-Wallis Test 0.44; ^5 ^A trend towards a deviation from Hardy-Weinberg equilibrium was observable for genotype frequencies for Δ BMI-SDS (exact p = 0.02). This finding, however, is not surprising and expected in case of a true genetic association and indeed the number of homozygotes for the at-risk A-allele was increased in these patients compared to controls.

**Table 3:** Baseline measures of blood parameters and BMI-SDS in German fasted obese children and adolescents

Parameter	N^1^	Genotype	N (%)	Mean ± SD	Additive genetic model^2^		
					
					Estimate	95% CI	p-value^3^
BMI-SDS^7^	519	TT	140 (27)	2.53 ± 0.49	0.025	-0.034...0.083	0.410
		AT	238 (46)	2.60 ± 0.51			
		AA	141 (27)	2.58 ± 0.52			

TGL [mg/dl]^4^	332	TT	94 (28)	115.15 ± 65.17	-0.008	-0.039...0.022	0.590
		AT	159 (48)	107.89 ± 52.75			
		AA	79 (24)	109.52 ± 53.64			

LDL [mg/dl]^5^	323	TT	93 (29)	104.18 ± 30.12	-0.001	-0.021...0.020	0.947
		AT	153 (47)	106.52 ± 32.67			
		AA	77 (24)	103.42 ± 30.75			

HDL [mg/dl]^6^	324	TT	93 (29)	50.25 ± 11.26	-0.004	-0.018...0.010	0.601
		AT	153 (47)	50.92 ± 11.28			
		AA	78 (24)	49.33 ± 11.54			

Glucose [mg/dl]^7^	480	TT	136 (28)	85.54 ± 9.09	-0.003	-0.009...0.003	0.350
		AT	216 (45)	84.67 ± 9.54			
		AA	128 (27)	84.37 ± 8.60			

^1 ^total number of obese individuals, from which baseline measures were available; ^2 ^linear regression analyses for log10-transformed parameters or BMI-SDS  including covariates age and sex; ^3 ^two-sided p-value; ^4 ^TGL: triglycerides; ^5 ^LDL: low density lipoprotein; ^6 ^HDL: high density lipoprotein; ^7^A trend towards a deviation from Hardy-Weinberg equilibrium was observable for genotype frequencies for BMI-SDS and glucose (exact p = 0.07; 0.03, respectively). This finding, however, is not surprising and expected in case of a true genetic association and indeed the number of homozygotes for the at-risk A-allele was increased in these patients compared to controls.

## Discussion

The case-control study comprising 519 German overweight and obese children and adolescents and 178 normal weight adults underscores the robust association of the rs9939609 A-allele with early onset obesity [1,2]. However, rs9939609 has, amongst other *FTO *SNPs, also recently convincingly been shown to be associated with adult obesity [2,3]. A GWA based on the isolated population of Sardinia further revealed that the association of SNP rs9939609 is even higher for increased hip circumference than for BMI (p = 1.3 × 10^-7 ^and 1.8 × 10^-6^, respectively) [3]. However, as waist circumference was not reported in this study, it remains unclear whether genetic variation of *FTO *play also an important role in the development of the metabolic syndrome. After correcting for BMI, however, no residual association of SNP rs9939609 has been found for other phenotypes, like type 1 diabetes mellitus (T1DM) [11], and T2DM [2,12]. Despite a small trend of the risk A-allele carriers to lose less weight than the non-risk allele carriers, the follow-up study comprising 207 German overweight and obese individuals revealed no significant association of the rs9939609 genotype with body weight loss due to an obesity intervention program. This finding is in accordance to previous results showing no association of the *FTO *SNP rs8050136 with weight loss [5]. Similarly, analyses of several fasting blood parameters (glucose, triglycerides, LDL and HDL cholesterol) revealed no association with rs9939609 within our group of young overweight and obese individuals. As indicated in a recent publication [13] the genetic effect sizes for these traits might even be smaller than those for the repeatedly observed effect of rs9939609 on the BMI. Thus, considerably larger sample sizes will be necessary to detect an effect of rs9939609 on BMI related traits.

It is noteworthy, that several SNPs in intron one of *FTO *are in strong linkage disequilibrium (LD) with rs9939609 [1-4]. It is currently unclear which of these variants is functionally relevant and thus underlying the repeatedly observed associations to obesity.

The biological function of *FTO *is largely unknown. However, *FTO *is expressed in multiple tissues throughout the brain and the periphery with high expression in the pituitary and adrenal glands and the hypothalamus [4,14]. It has thus been suggested that FTO might play a role in the hypothalamic-pituitary-adrenal axis [4]. Very recently, it was further shown that *Fto *mRNA levels were reduced by 60% in the arcuate nucleus of *WT *mice following food deprivation and that this decrease of *Fto *mRNA was independent of the fasting induced decrease of leptin levels [14]. Further analyses revealed that *FTO *shares sequence motifs with Fe(II)- and 2oxoglutarate (2OG)-dependent oxygenases [14,15] and that murine Fto catalyzes the Fe(II)- and 2OG dependent demethylation of 3-methylthymine in single stranded DNA with concomitant production of succinate, formaldehyde and carbon dioxid [14]. Localization of Fto to the nucleus in transfected cells [14] is consistent with this potential role of Fto in nucleic acid demethylation. Additionally, allelic variation of *FTO *has recently been shown to be associated with a reduced cerebrocortical insulin effect on beta activity [16]. It is therefore possible that impaired cerebrocortical insulin response at least partly accounts for the implication of *FTO *variants on obesity [16]. One might also consider that the association of the variants in intron one of *FTO *points to an obesity gene in the vicinity of the associated SNPs. *KIAA1005 *is 61 kb upstream of the 5'end of *FTO *and is transcribed in the opposite direction [2]. However, there is currently no indication that *KIAA1005 *has any functional implication on body weight related traits. In summary, we confirmed the association of the rs9939609 obesity risk A-allele with overweight and early onset obesity. We found no significant association of body weight reduction or any of the analysed blood parameters with the rs9939609 risk genotypes within our group of young overweight and obese individuals.

## Conclusion

We conclude that variation in the first intron of *FTO *is a risk factor for early onset obesity while its impact on weight loss or serum levels of blood glucose, triglycerides and cholesterol (LDL, HDL) is not detectable in our study group. Thus, standardized effect sizes for the investigated quantitative traits are seemingly well below 0.25 (in units of standard deviations for each copy of the risk allele) as our study was well powered to detect standardized additive effects of 0.25 (α = 0.05; two-sided). Studies in larger sample groups will be necessary to ensure that genetic variation of *FTO *has indeed no effect on weight changes after a lifestyle intervention or on markers of glucose and lipid metabolism.

## Competing interests

The authors declare that they have no competing interests.

## Authors' contributions

TDM carried out the molecular genetic studies, participated in design and interpretation of data and drafted the manuscript. AH and JH conceived the design and participated in its design and coordination; helped to draft the manuscript and revised it critically. AS and TTN performed the statistical analysis and helped to draft the manuscript under supervision of HS. FS and CLR have made substantial contributions to the acquisition of data and revised the manuscript critically. TR has made substantial contributions to the acquisition of data, made substantial contributions to conception, design and interpretation of data and revised the manuscript critically. All authors read and approved the final manuscript.

## Pre-publication history

The pre-publication history for this paper can be accessed here:


